# Sensory Processing and Community Participation in Autistic Adults

**DOI:** 10.3389/fpsyg.2022.876127

**Published:** 2022-06-02

**Authors:** Nancy Bagatell, Dara V. Chan, Ya-Cing Syu, Elena M. Lamarche, Laura G. Klinger

**Affiliations:** ^1^Division of Occupational Science and Occupational Therapy, Department of Allied Health Science, University of North Carolina at Chapel Hill, Chapel Hill, NC, United States; ^2^Division of Clinical Rehabilitation and Mental Health Counseling, Department of Allied Health Science, University of North Carolina at Chapel Hill, Chapel Hill, NC, United States; ^3^TEACCH® Autism Program, University of North Carolina at Chapel Hill, Chapel Hill, NC, United States; ^4^Department of Psychiatry, University of North Carolina at Chapel Hill, Chapel Hill, NC, United States

**Keywords:** community participation, sensory processing, autism, adults, geographic positioning system

## Abstract

**Background:**

Sensory processing differences have been shown to impact involvement in community activities. However, relatively little is known about how these differences affect community participation in autistic adults.

**Objective:**

The objective of this study was to explore how sensory processing patterns of autistic adults impact community participation, including where people go, what they do, the amount of time in the community, and preferred locations.

**Methods:**

We used data gathered from six autistic adults and their caregivers who participated in two studies. From Study 1, we reviewed results of the Adolescent and Adult Sensory Profile (AASP) and transcripts from interviews with caregivers. From Study 2, we reviewed GPS tracking data and transcripts from structured interviews with autistic adults focused on community participation. We read transcript data, identified quotes related to sensory processing and community participation and constructed individual participant narratives which linked findings from interviews, AASP, and GPS tracking.

**Results:**

Participants included three males and three females ranging in age from 29 to 51. Each participant had a unique sensory processing profile that influenced where they went, the activities in which they engaged, how much time they spent in the community, and their preferred locations. Those whose sensory processing patterns indicated sensory sensitivity and sensory avoiding described the experience of certain environments as overwhelming and fatiguing and thus spent less time in the community and visited fewer places than those with other sensory processing patterns.

**Conclusion:**

Results highlight the importance of sensory processing, especially as it impacts participation in the community. Sensory processing patterns should be considered along with other personal and contextual factors when assessing community participation and personal sensory processing patterns should be matched with activities and environmental demands.

## Introduction

Participating in community life, including work, school, leisure, and instrumental activities, such as doing errands and going to the gym, is considered an essential component of health and wellbeing for individuals across the life span (Khetani et al., 2013; Kuykendall et al., 2015). Participation involves having access and opportunities to meaningfully and actively engage in activities and connect with others (Hammel et al., 2008). In the International Classification of Health, Function, and Disability model (ICF; World Health Organization, 2010), both person and environment (contextual) factors contribute to participation. Despite its importance, literature consistently reflects that people with disabilities have lower rates of community participation than people without disabilities (Verdonschot et al., 2009; Askari et al., 2015). This is particularly true for autistic individuals.^1^ Studies consistently indicate that autistic children and youth participate in fewer activities with less frequency than typically developing peers (Hilton et al., 2008; Potvin et al., 2013; Egilson Snæfrídur et al., 2017). This pattern of reduced community participation has been found to continue into adulthood. A longitudinal review of the National Longitudinal Transition Study-2 (NLTS-2) data reported a significant decrease in community participation from adolescence to adulthood, with one community activity per year considered a positive result (Myers et al., 2015). Autistic adults also report being less satisfied with their participation than typical adults (Song et al., 2021). Additionally, despite interest in activities in the community, autistic adults report they do not actually participate in these activities (Shea et al., 2021).

Relatively little is known about the determinants or predictors of these limited patterns of participation. Studies that do address the determinants of reduced participation suggest a confluence of many factors. Song et al. (2021) noted how environmental factors such as access to services and type of residential setting influenced community participation. Chan et al. (2021) found autistic adults who had a higher density of bus stops within a half mile of their home location had higher rates of volunteering, getting together with friends, and being invited to activities with friends. Additionally, in a scoping review of the literature on the participation of autistic children and youth, Askari et al. (2015) noted that environmental factors such as family support and social attitudes, the social and communication demands of the activity, and the clinical characteristics of autism such as restricted interests, challenging behavior, and sensory processing differences have been reported to impact community participation.

Sensory processing, the ability to register and modulate sensory information and respond to environmental demands, is a fundamental component of everyday life. It is through our senses that we interpret, experience, and respond to life events. Each person has a unique way of processing sensory information based on their nervous system, life experiences, and cultural values and beliefs. Sensory processing differences are typically described as either sensory hypersensitivity (a low neurological threshold) or sensory hyposensitivity (a high neurological threshold) which result in unique behavioral responses and preferences. According to Dunn (1997), people may seek out sensory input, avoid sensory input, have difficulty detecting sensory input, or have greater sensitivity to sensory input.

Sensory processing differences are commonly reported in autistic children, with prevalence rates of 56.8–92.5% (Dellapiazza et al., 2021). Compared to neurotypical children, autistic children demonstrate more difficulty filtering sensory stimuli and regulating responses to sensory input, such as being easily distracted by background noise or having increased sensitivity to tastes and textures of food (Tomchek and Dunn, 2007; Tomchek et al., 2014). These unique sensory processing behaviors may limit participation in social and recreational activities (Hochhauser and Engel-Yeger, 2010; Reynolds et al., 2011) and impact family activities and routines with families avoiding going places and attending events in the community that do not fit with their child’s sensory preferences (Schaaf et al., 2011; Bagby et al., 2012). In the ICF model, the individual’s sensory processing pattern, a person factor, interacts with the sensory environment of locations visited in the community. Participation is limited when sensory preferences do not match the sensory stimuli of the environment where desired community activities occur.

Sensory processing differences are also common in autistic adults, with prevalence rates ranging from 77 to 95% (Crane et al., 2009; Gonthier et al., 2016). Studies examining sensory processing profiles suggest that patterns may vary. For example, using sensory processing survey measures, Tavassoli et al. (2014) and Syu and Lin (2018) reported more overresponsivity in autistic adults without intellectual disability, while Crane et al. (2009) noted more diverse sensory patterns in this population. Additionally, Gonthier et al. (2016) reported lower registration behaviors and less sensory seeking behaviors in autistic adults with an intellectual disability. The impact of sensory processing patterns on everyday life has also been explored in studies using qualitative methods. In these studies, autistic adults describe how their participation in the community is affected by sensory experiences, such as being unable to go to nightclubs with friends or being distracted by colors of signs in the workplace (Robertson and Simmons, 2015; Clince et al., 2016). To date, no study has explicitly explored how autistic adults’ sensory processing patterns influence community participation, specifically where people go, how often they are in the community, and their preferred activities and locations. This study offers a novel approach by integrating qualitative interviews, quantitative surveys, and Geographic Positioning System (GPS) tracking to understand the impact of sensory processing on community participation in autistic adults.

## Materials and Methods

In this paper, we report on data gathered from six autistic adults and their caregivers who participated in two different studies. Both studies were approved by the university Institutional Review Board.

### Study 1

Study 1 was a long-term follow-up study to assess adult outcomes of individuals who were diagnosed with autism as children between 1969 and 2000 at a university-based autism center. Participants (n = 55) completed a battery of assessments including IQ (Stanford Binet 5) and adaptive behavior (Vineland Adaptive Behavior Scales 2; VABS-2). Caregivers completed an interview focused on services and future plans and the Adolescent and Adult Sensory Profile (AASP). For this paper, we used demographic information and full-scale IQ (FSIQ) to describe our participants and the results of the AASP and transcripts from the VABS and caregiver interview for further analysis. These data were collected from 2013 to 2016.

### Study 2

Participants for Study 2 were recruited from Study 1. Study 2 was a mixed methods study focused on assessing community participation using GPS tracking with 23 autistic adults over a 1-week period. After the study tracking week, participants completed a follow-up visit to review the GPS maps created and participated in a structured interview regarding community activities, barriers to participation, and the importance of different locations visited. These interviews were recorded and transcribed verbatim. Data were collected from 2016 to 2017.

The samples of the two studies were compared to identify individuals who participated in both studies. This comprised a sample of 10 individuals.

### Measures

#### Adolescent/Adult Sensory Profile

The Adolescent/Adult Sensory Profile (AASP; Brown and Dunn, 2002) is a 60-item questionnaire which assesses behavioral responses to sensory experiences in everyday life. The questionnaire is for individuals ages 11 to 65. It is based on Dunn’s (1997) sensory processing model. There are two key constructs in this model: neurological thresholds and self-regulation. One’s neurological threshold, the point at which one notices and responds to sensory stimuli, can range from low to high. Self-regulation is also on a continuum, with behavioral responses ranging from passive to active. When these continua intersect, sensory processing patterns can be identified as: low registration, sensation seeking, sensory sensitivity, and sensation avoiding. Items on the AASP reflect the following sensory categories: taste/smell, touch, movement, auditory, visual, and activity level. Respondents rate the frequency with which they respond to each item using a 5-point scale (1 = almost never, 2 = seldom, 3 = occasionally, 4 = frequently, and 5 = almost always). This results in a total score for each quadrant ranging from 15 to 75. Higher scores indicate a higher frequency of each sensory processing pattern. Based on raw scores, sensory processing patterns are described as: much less than most people (2% of the population); less than most people (14% of the population); similar to most people (68% of the population); more than most people (14% of the population); and much more than most people (2% of the population; Brown and Dunn, 2002). The AASP is a reliable and valid tool which has been used in other studies to assess sensory processing in autistic adults (Crane et al., 2009; Horder et al., 2014).

#### Community Participation

**Figure 1:**
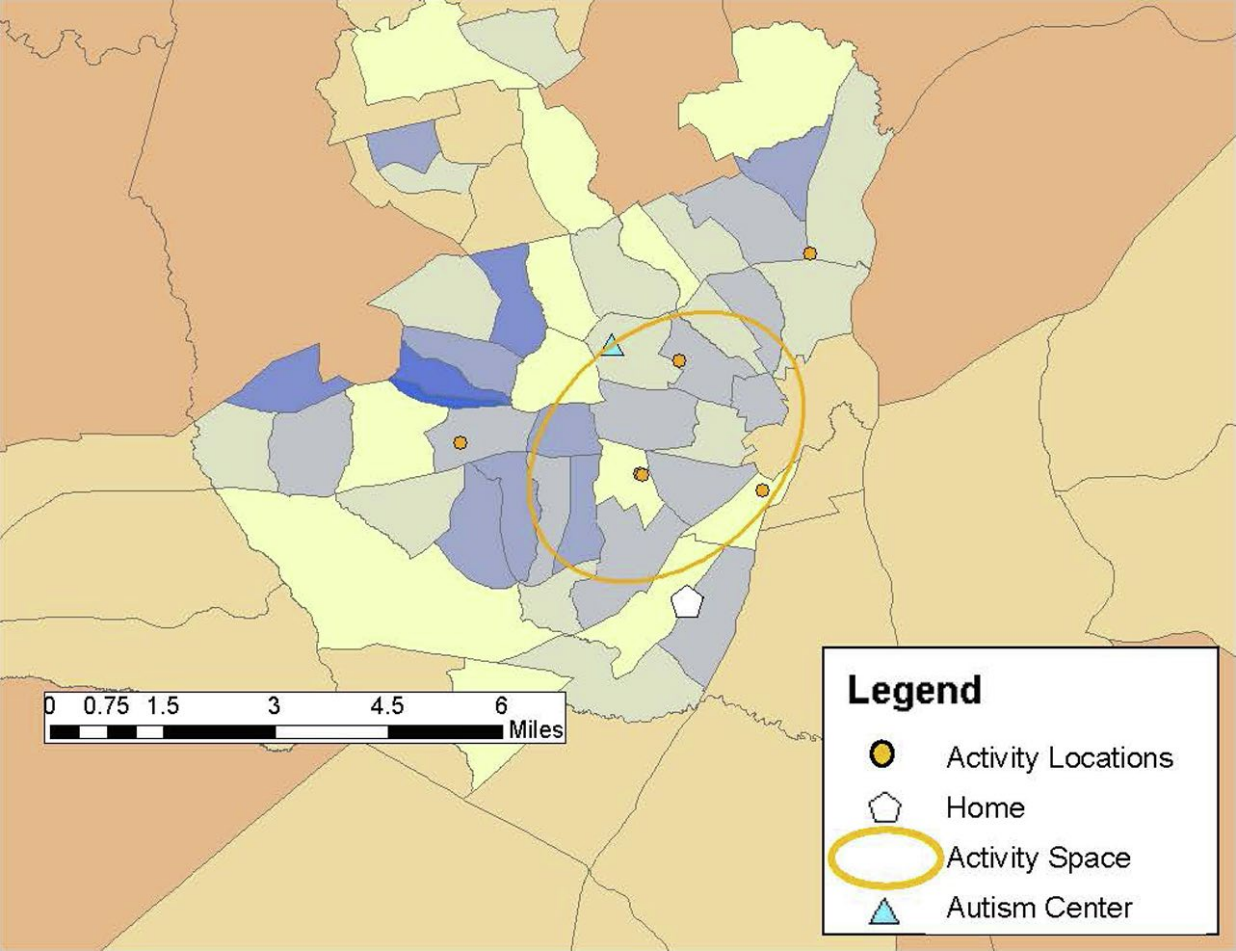
Steve’s activity space (23.45 mi^2^) reflecting visits to a few locations dispersed throughout his community area during the 1-week study period.

**Figure 2:**
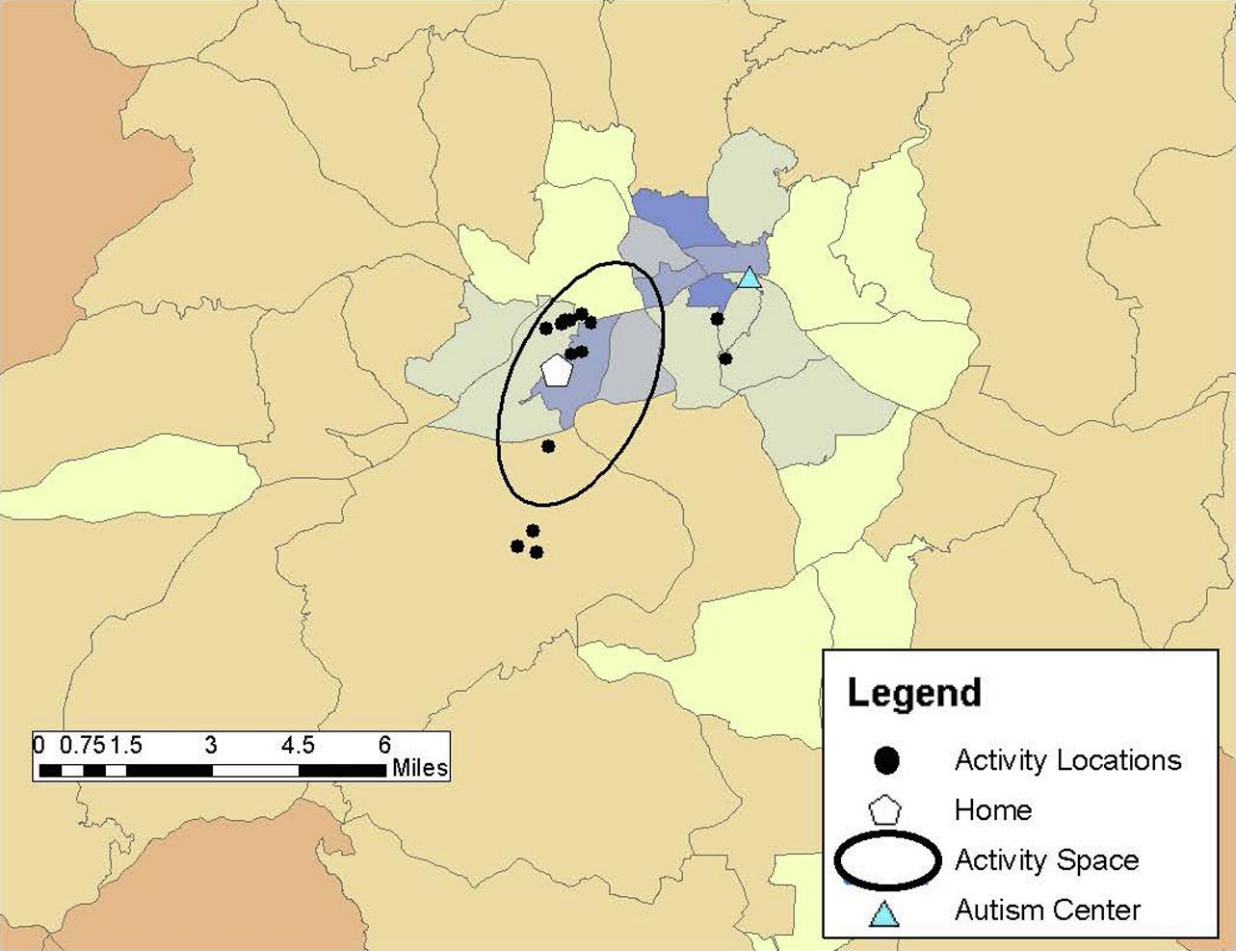
John’s activity space (8.51 mi^2^) based on visiting several locations clustered close to his home and to each other during the 1-week study period.

**Figure 3:**
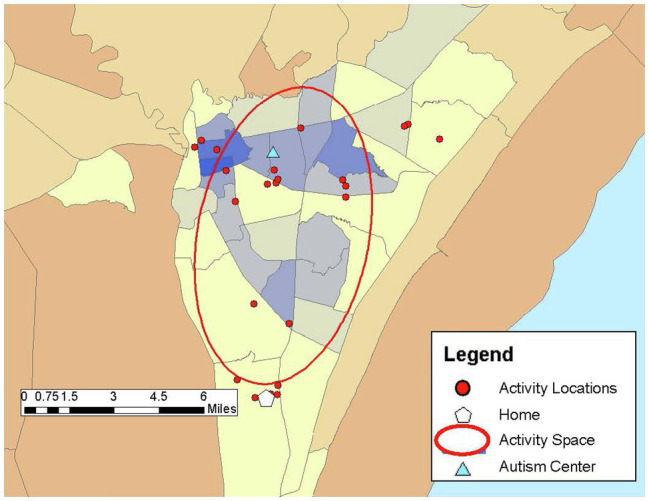
Sherri’s activity space (45.22 mi^2^) shows both the wide dispersement and a large number of locations visited throughout her community area during the 1-week study period.

**Figure 4:**
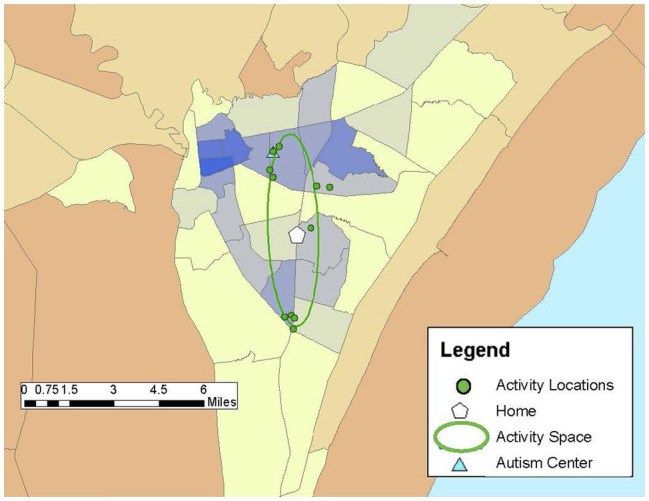
Pete’s community locations and activity space (8.6 mi^2^) from the GPS study week reflect his visits to several locations close to home and the autism center he volunteered with during the 1-week study period.

**Figure 5:**
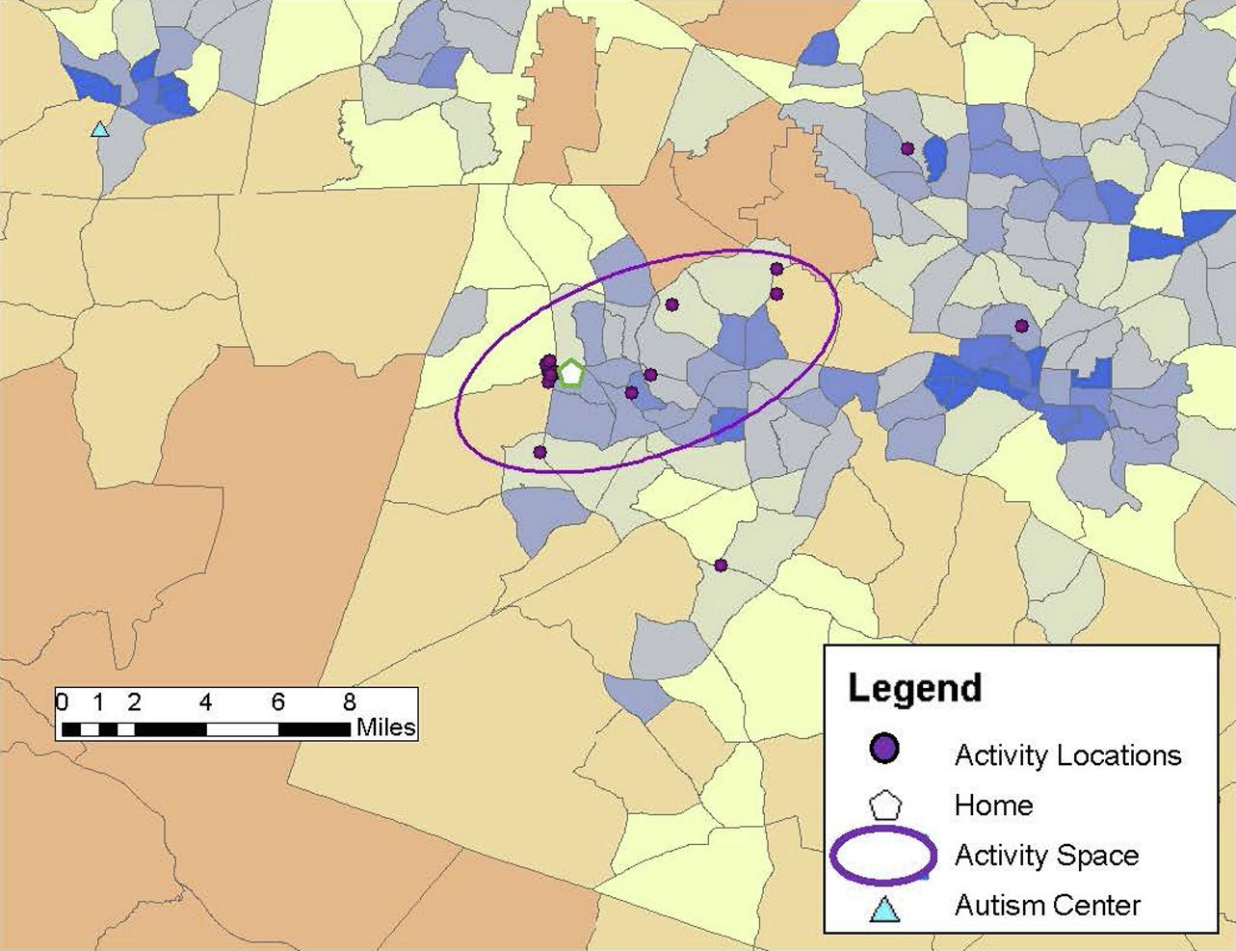
Patti’s activity space (44.72 mi^2^) based on locations visited in the community during the 1-week study period, reflecting a combination of activities clustered around her home and those requiring greater geographic mobility.

**Figure 6:**
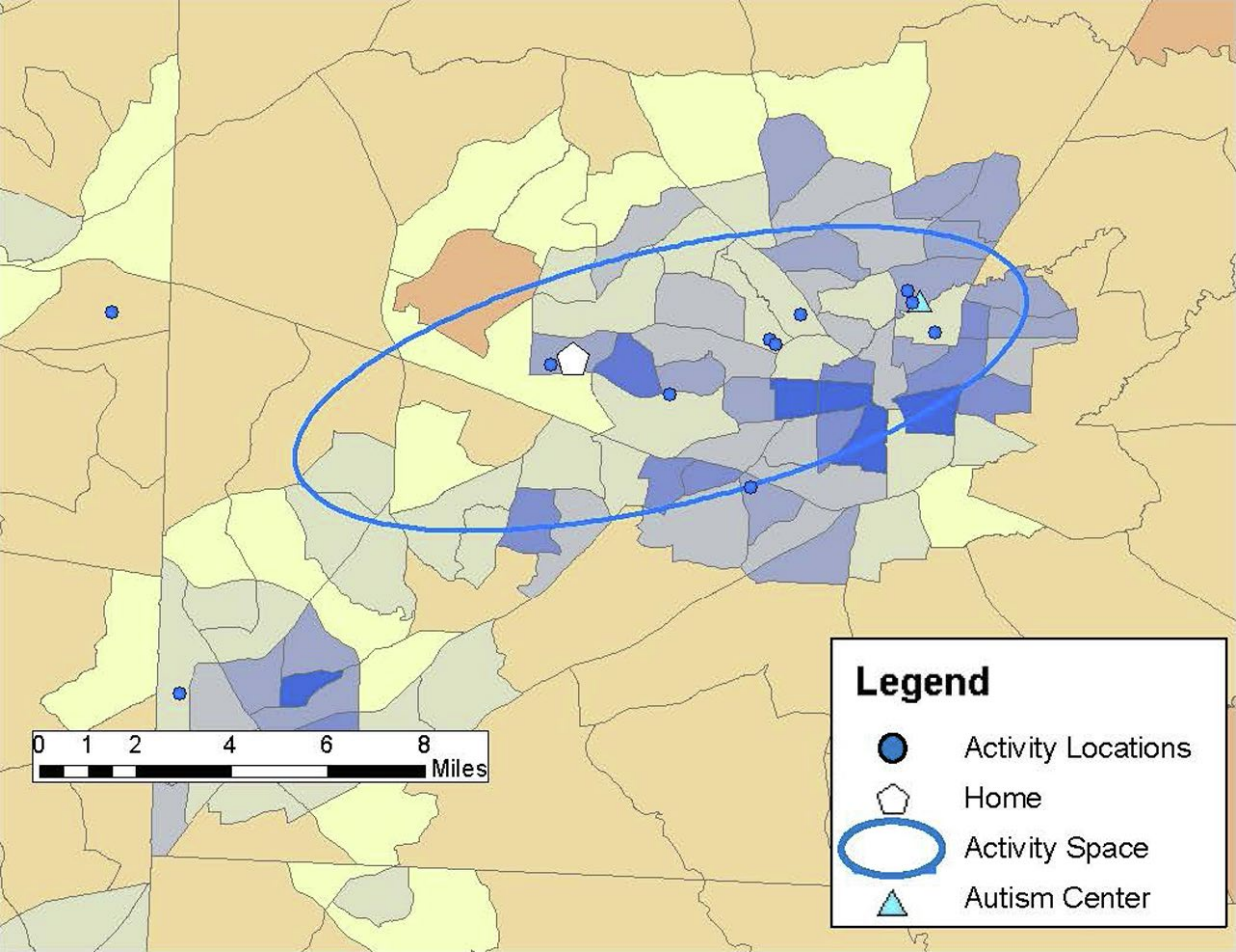
Beth’s activity space (64.51 mi^2^) showing the wide dispersion of community activity locations from the GPS study week, particularly to visit her family in another town on the weekend.

Drawing on previous research (e.g., Hordace et al., 2014; Brusilovskiy et al., 2016), community participation was measured through the GPS tracking data collected over the 1-week study period and qualitative data from the follow-up structured interview in Study 2. Participants carried PocketFinder GPS trackers which recorded latitude/longitude coordinates of their location in the community every 2–5 min. Participants (or participants and caregivers) completed daily travel diaries during the 1-week period, providing more context to the locations visited such as the purpose of the activity, whether the activity was done alone or with others, and transportation used. From the GPS data, number of unique locations visited in the community, time spent away from home, and activity space size were examined as primary outcome measures of participation. Activity space was calculated as a 1 standard deviation ellipse using ArcGIS mapping software to incorporate the distance from one’s home to the community locations visited during the study week, representing individuals visit some, but not all locations each day (see Figures 1–6).

In addition to the GPS data, transcripts of the structured interviews with the autistic adults were reviewed. Questions of particular interest for the current analysis included as: “Can you tell me about any barriers you faced this week to participating in activities outside of your house?” and “Looking at the map and all of the places that you visited this week, including your home, which places are most important to you? Why?”

### Data Analysis

The analysis process for the current investigation involved creating a participant narrative linking sensory processing patterns to community participation. The study team met to examine potential patterns identified on the AASP in conjunction with GPS tracking measures, activity space maps, and summary interview data of key questions. Each member of the research team then was assigned two to three participants to complete an in-depth review of the available study data, including reviewing the transcripts from the VABS-2 and caregiver interview from Study 1 and the participant interviews from Study 2. Team members highlighted any content that appeared to be related to the impact of sensory processing in daily activities and community participation. The study team met again to share the findings of these in-depth reviews and selected six participants who had rich data and reflected a range of sensory processing patterns and demographic characteristics. Team members then returned to the data and did a careful review of the transcripts to ensure information related to sensory processing and community participation was not overlooked. The team then met to specifically discuss how information obtained from the GPS tracking (time away from home, number of locations, and activity space) related to sensory processing and community participation. Reviewing and analyzing data multiple times and discussing analytic insights with team members enhanced the rigor of our analytic process. The findings from these six participants are the focus of this paper.

## Results

Participants included three males and three females ranging in age from 29 to 51 (see Table 1 for complete demographic information). Participant living situation and employment status varied, and only one drove independently. Each participant had a unique sensory processing profile (see Table 2) that may have influenced where they went, the activities in which they engaged, how much time they spent in the community, and their preferred locations, as illustrated through the case descriptions that follow.

**Table 1:** Demographic information.

Case	Age	Gender	FSIQ	Race	Living status	Employment	Transportation
Steve	32	M	60	Asian/African American	Family	None	Parents
John	33	M	104	White	Family	None	Family
Sherri	29	F	80	White	Group Home	Part time	Others
Pete	49	M	77	White	Family	Part time	Drives
Patti	31	F	78	White	Apartment (Roommate)	Part time	Bus, others
Beth	51	F	40	White/American Indian	Group Home	None	Others

**Table 2:** Sensory processing patterns and GPS tracking data.

Case	Low registration	Sensation seeking	Sensory sensitivity	Sensation avoiding	Unique locations	Time away (Hours:Mins)
Steve	Similar to Most People	*Much Less Than Most People*	*Much Less Than Most People*	*Much Less Than Most People*	7	02:01
John	*Much More Than Most People*	*Less Than Most People*	*More Than Most People*	*Much More Than Most People*	14	01:22
Sherri	Similar to Most People	Similar to Most People	Similar to Most People	*More Than Most People*	25	08:01
Pete	Similar to Most People	Similar to Most People	*Less Than Most People*	Similar to Most People	11	05:48
Patti	*More Than Most People*	Similar to Most People	Similar to Most People	Similar to Most People	17	05:03
Beth	Similar to Most People	*Less Than Most People*	*More Than Most People*	*Much More Than Most People*	11	03:46

*Steve: “We have to drag him out when it’s time to go!”*

Steve was a 32-year-old African American/Asian male who lived with his family, which included his niece, nephew, and cousin, with whom he spent a lot of time. He reported he has always lived at this family residence. He was not employed and relied on his parents for transportation. His FSIQ was 60. Steve communicated easily and was friendly and outgoing during the research process. Steve spent most of his time during the study week at home since his mother, his primary form of transportation, was ill. However, he was “out and about” with his family an average of 2 hours a day running errands, transporting other family members, and going to fast-food restaurants. He enjoyed interacting with people in the community and noted that he sometimes “gives them a hug.” Although Steve’s mother noted his community activities were less during the data collection week than during a typical week, Steve visited seven different places with an activity space of 23.45 mi^2^ (Figure 1) and spent most of his “out and about” time in the car. Typically, Steve spent time playing basketball at the community center and enjoyed ice skating and participating in Special Olympics. His mother indicated that Steve also enjoyed parties at the community center, and that he loved music and dancing. She recounted, “We have to drag him out when it’s time to go!” A favorite activity that occurred once a year was attending the State Fair where Steve enjoyed going on rides and eating different foods. He indicated that his most important place was the mall.

Steve’s scores on the AASP suggest that his registration is in the typical range, his sensation seeking is less than others, his sensory sensitivity is much less than others, and his sensation avoidance is much less than others. Overall, Steve’s sensory processing pattern supported his participation in activities in the community and enabled him to visit sensory-rich environments, interact with people in the community, and enjoy participating in activities such as sports and dancing that provided movement experiences. Steve’s community participation was not limited by his sensory processing patterns but rather was constructed to fit with his family’s routines and activities.

*John: “I usually deal with a lot of things online.”*

John was a 33-year-old, White male who lived with his grandmother. He reported living in his current residence for about 18 years. He was not employed and relied on his grandmother for transportation. Although he was able to drive himself, he described it as a scary activity that “plays with my senses.” His FSIQ was 104. John communicated readily, though had a slight tendency to mumble at times. He spent most of his time at home, with an average of just over an hour away from home each day. He had an activity space of 8.51 mi^2^ (Figure 2). His community activities for the GPS tracking week centered around picking up food at a series of drive-through, fast-food restaurants. He also ran errands with his grandmother as he described that she did not like to go places by herself, and had a few medical related locations based on a recent toe injury. He indicated his favorite place was being home, preferring the more controlled environment of online social interactions. He stated, “I usually deal with a lot of things online, so I do not usually have to worry much about meeting people face to face or whatever…” When asked if there were any places he wished he could spend more time, John noted:

I’m content with the social interaction I get, as I said, that most of my social interactions are online. And that matters more to me than like in-in person because of the shield of anonymity it provides… because we can be who we want to be on the [inter]net, for the most part.

John’s scores on the AASP suggest that his sensory registration is higher than others, his sensation seeking is less than others, his sensory sensitivity is slightly more than others, and his sensation avoidance is much more than others. Overall, John’s sensory processing pattern supports his preference to stay home and limit his participation in activities in the community, consistent with the GPS data. He noted he is content to engage in online social activities with his presence in the community mainly to pick up fast-food or assist his grandmother in running errands. However, he also reported he and his grandmother strategically plan their day every morning to map out how to complete community activities in the most efficient time as a desire to save money on gas, so financial considerations may also contribute to his low time away from home and limited community activities.

*Sherri: “I’m very much happy with stuff I am doing now.”*

Sherri was a 29-year-old White female who lived in a group home. She had been living in the group home for 6 years. She held two part-time jobs, working as a stock clerk at a retail pharmacy 2 days a week, and as a field trip assistant at a children’s museum 1 day a week. She relied on the group home staff workers or family members for transportation to various activities. Her FSIQ was 80. She often deferred to her staff member who was present during the GPS interview for confirmation of answers, or at times needed prompting by the staff member to be able to answer some of the questions. Sherri spent most of her time during the week away from the group home, spending an average of 8 hours a day in the community. She was involved in a number of activities, visited 25 unique locations during the study week, and had an activity space size of 45.22 mi^2^ (Figure 3). Sherri reported the drug store where she worked was the location most important to her. In addition to her two part-time jobs, she also volunteered at a food bank, a hospital, and a day program multiple times a week. Other activities included going grocery shopping, browsing in a bookstore, going to the bank, and attending practice for Special Olympics. She also walked for exercise and participated in a yoga class at the YMCA. Sheri spent time visiting friends and neighbors and went to her parent’s house. In addition to these activities that were part of her typical routine, she also engaged in several events during the study week that were special events, including attending a Special Olympics social event, a holiday party at the day program where she volunteered, and going out to eat at a restaurant and to a water theme park for her roommate’s birthday celebration. At times these activities occurred within the same day without breaks or returning home, but this did not seem to bother Sherri, who noted, “I had a great time [at work] and then we went back to the day center for the party.” She reported she had no barriers to participating in activities during the week.

Sherri’s scores on the AASP indicate she has no sensory processing concerns, which is consistent with her high engagement in a variety of community activities. Without sensory processing limitations, she was able to participate in both routine and non-routine activities during the week, including several social activities. Her sensory profile allowed for her involvement with a number of routine, scheduled activities arranged through her group home, but she was highly involved in social activities with others as well. Sherri was generally satisfied with her participation in the community. She stated, “I’m very much happy with stuff I am doing now,” but added she wished she could do more activities.

*Pete: “It just feels beautiful to feel.”*

Pete was a 49-year-old, White male who lived with his mother. He had lived in the same home his entire life. He had a FSIQ of 77 and communicated well though he had a slight stutter at times. Throughout a typical week Pete visited 11 unique places with an activity space of 8.6 mi^2^ (Figure 4) and frequently drove himself to these locations. He spent more than half his day away from home (averaging nearly 6 hours per day) but chose locations that were close to home. Pete worked part-time doing light janitorial work and volunteered at several community locations including the library and two local service agencies. He had several favorite restaurants and cafes that he regularly visited for lunch after working or volunteering. Pete noted that at one café the barista knew him so well that “when she sees me coming, pulling up in the parking lot…she prepares for me either an Incrediberry smoothie or a 12-ounce latte.” He participated in grocery shopping with his mother, and he was active at church through weekly attendance at religious services, a monthly prayer breakfast, and singing with the choir. In addition to the places visited during the GPS study week, he reported he enjoyed going to music and bookstores around his local community and taking walks outside around his home and community. He loved to be at the beach saying, “It just feels beautiful to feel, just feels beautiful to hear, hear the ocean feel…that ocean breeze blowing.” Spending time with family was also an important activity for Pete. He indicated his most important place was his local autism agency, which had been a part of his life since he was a preschooler, and he noted his “whole life is centered around the program itself.”

On the AASP, Pete’s scores indicated no concerns with sensation avoidance or sensation seeking; his registration was within the typical range but approaching higher than typical, and his sensory sensitivity was lower than the typical range. While he worked and volunteered across four locations throughout the week, at each place he engaged in highly repetitive tasks (cleaning, paper shredding, copying, book shelving). This daily pattern may reflect a need for variability in the location and daily tasks (high registration), yet his low sensation sensitivity allowed him to focus on repetitive tasks with less distraction from outside stimuli. Pete was able to construct a set of weekly activities and engage meaningfully across all the community locations he visited that met his sensory needs and where he felt personally fulfilled.

*Patti: “The worst thing you can do to her is take away an activity.”*

Patti was a 31-year-old White female who lived in a supervised apartment in an urban area with a roommate who also had a disability. She had lived in the apartment for 6 years. Patti worked part time at a retail drug store 2 days per week and was involved in several activities in her community area, often traveling great distances to these activities. To traverse the community, she rode the bus independently to routine locations, such as to work or to get fast-food; otherwise, she received rides from apartment staff, a personal support, or her parents. Her FSIQ was 78 and she communicated easily, although high levels of anxiety were apparent at times during the interview through the use of repeated questions. Patti was very active in the community during the week, visiting 17 unique locations and averaging 5.5 hours per day away from her apartment, with an activity space of 44.72 mi^2^ (Figure 5). During the study week, Patti spent time in several activities that were part of her regular routine, including attending a day program, drama and dance classes, going to the YMCA and taking walks in her neighborhood for exercise, and visiting her parent’s house on the weekend. She also went to the bank, grocery shopping, and picked up fast-food. In addition, she participated in a social activity sponsored by her supportive housing. Although not part of the study week, Patti noted the mall was one of the most important places to her: “I love to shop for things. And go to the arcade.” Routines and schedules were very important to Patti, as she noted she was very comfortable in her apartment since it allowed her to keep her routines.

Patti’s sensory profile on the AASP showed she had low registration, suggesting that she may miss sensory input and therefore not be affected in situations with high sensory stimuli. Her sensory processing profile was consistent with her ability to tolerate activities in a variety of environments as she participated in many activities in the community. In the caregiver interview, her parents shared, “She loves activities, yes. The worst thing you can do to her is take away an activity.” However, it is noteworthy that these activities were part of her regular routine, which could be consistent with individuals with low registration. In the interview with her parents, they shared that Patti has high anxiety and obsessive–compulsive disorder, and often engages in her routines for self-soothing and to create predictability in her life. Her parents also noted, “She’s loud. Very loud. Extremely loud, [and] does not realize when she’s being loud,” which may reflect her low registration of her own auditory output. A low registration pattern, often associated with not noticing sensory stimuli, may have contributed to her parent’s concerns with her eating behavior, as they shared in the caregiver interview:

She eats too much food too fast, she talks when she, ugh it’s horrible. Her table manners are like … she’s not really aware, just like with her loud talking, that, that there’s food dropping and stuff.

However, they also noted the impact of her anxiety that may contribute to her lack of external awareness: “It’s that her anxiety, you know, kind of keeps her focused on herself and her needs.”

*Beth: “She does not like loud environments.”*

Beth was a 51-year-old White/American Indian female who had lived in her current group home for 27 years. She was not employed and relied on others for transportation. Her FSIQ was 40, and she had communication challenges, often repeating sounds during the GPS interview or clapping hands and vocalizing when she was asked questions. Though Beth was fairly social, she did not want people to hug or touch her. On weekdays, Beth spent most of her day at a day program and usually went for a ride with the group home staff and peers for an average of 4 hours per day. Beth went to her sister’s house every Friday and stayed for weekends. During the study week, Beth visited 11 different places (activity space of 64.51 mi^2^, Figure 6) with group home staff or her family, including activities of shopping, exercising, and dining. According to her siblings, besides these typical activities, she also enjoyed her time at the music center and church, especially when they had musical programs, because of her love for music. The most important places to Beth were her siblings’ houses. She was comfortable at both places and happy to stay with the family, sometimes watching concerts together on television. However, Beth’s siblings mentioned her dislike of loud sounds was a barrier to community participation as they noted, “We cannot take her anywhere real loud,” and “She does not like loud environments, a lot of activity.” The family carefully chose restaurants and shops they visited to prevent Beth from sensory overload; they stated that “the big Walmarts get on her nerves…that’s a lot of stimulation there.”

Beth’s scores on the AASP suggested that her registration was in the typical range, her sensory seeking was less than others, her sensory sensitivity was more than others, and her sensation avoidance was much more than others. Beth’s sensory processing pattern may impact her participation in the community and contribute to her avoiding sensory-rich environments and being physically intimate with others. Overall, Beth’s participation in the community appeared to be related to not only her sensory processing patterns but also how her days were constructed by the group home and her family.

## Discussion

The profiles presented highlight the importance of sensory processing in daily life, especially as it impacts participation in the community for autistic adults. While recent studies have noted how contextual factors such as residential setting, geographic location, and availability of services affect community participation patterns (Chan et al., 2021; Song et al., 2021), this is, to our knowledge, the first study to examine sensory processing patterns in conjunction with GPS tracking, travel diary data, and semi-structured interviews, which provided information on barriers to and satisfaction with community participation. Examining multiple sources of data revealed individuals whose families reported their autistic adult had extreme sensation avoiding profiles, such as John and Beth, spent less time in the community during the study week, while those who did not have sensory sensitivity or high sensation avoiding, such as Sherri, Pete, and Patti, spent more time participating in activities in the community. Although Steve had the lowest sensory sensitivity and sensory avoidance scores of the group, his GPS data was more similar to John and Beth, with less time spent in the community. His limited participation, however, was attributed to his mother being sick during the study week, reflecting a tangible example of the often-reported relationship between community participation and access to transportation (Badia et al., 2011; Lubin and Feeley, 2016), and underscores its importance.

The findings from our study may also provide context for past community participation research. Using survey data comparing community participation between autistic adults and those in the general population, Song et al. (2021) noted significant differences in the amount of participation as well as the types of community activities rated as important. However, the authors note these findings may be more indicative of barriers to participation rather than participation preferences (Song et al., 2021). The interviews with the autistic adults revealed participants were participating in activities and environments they reported as important to them. Notably, those whose sensory processing patterns did not include extreme sensation avoiding or sensory sensitivity (Steve, Sherri, Pete, and Patti) all identified locations in the community where they engaged in desirable activities as the most important locations to them, while John and Beth, who both were high in sensory sensitivity and sensation avoiding, reported the familiarity, comfort, and perhaps predictability of the family home environment as their favorite location. Combining GPS and participant and caregiver interview data with sensory processing profiles allowed a more comprehensive view of how these sensory preferences may impact community participation.

The current study also contributes to the sensory processing literature confirming that sensory processing differences continue into adulthood and may influence how individuals engage with their environments. Autistic adults may either actively manage sensory responses to the environment or may not attune to cues in the environment that may promote participation (Crane et al., 2009; Tavassoli et al., 2014; Gonthier et al., 2016; Syu and Lin, 2018). The current findings contribute to the literature by suggesting that certain locations in the community may present sensory environments that limit participation for autistic adults, for example, environments that are noisy or unpredictable. Preferences for participation in specific community activities may also be shaped by one’s sensory processing profile. As noted above, in the cases of Sheri, Patti, and Pete, their sensory processing profiles, which were largely “similar to most people,” allowed participation in a variety of environments. In contrast, John intentionally chose to limit his time in the community, preferring the home environment and online social interactions that fit with his sensory processing profile characterized by higher sensory avoidance. Similarly, Beth’s family was mindful of seeking environments that did not cause her distress based on her heightened sensitivity to sensory input, as reflected in her sensory processing profile.

In addition to considering the sensory aspects of locations in the community, findings highlight how sensory demands of activities in conjunction with environmental demands impact community participation. For example, Steve had a strong preference for activities that provided movement, such as basketball and dancing. Given his sensory profile characterized by “much less than most people,” Steve was able to participate in these activities in sensory stimulating environments in the community as well as in a more sensory controlled environment at home. However, given Beth’s sensory profile of heightened sensory sensitivity and sensory avoiding, Beth was not able to participate in listening to music in the community; however, she was able to enjoy music where the volume of sound and the density of people could be better controlled given her living situation.

Past survey research on the impact of living situation on community participation of autistic adults noted that adults who live with family members have less community participation than those living without family members (Dudley et al., 2019; Song et al., 2022). This may be due to family members being primarily responsible for planning community activities for individuals into adulthood (Levy and Perry, 2011; Gray et al., 2014), where often they need to prioritize managing daily living activities over social events (Cheak-Zamora et al., 2015). Family members may also wish to protect their autistic adult from negative community interactions based on past experiences and prefer keeping to home-based activities (Ryan, 2010; Song et al., 2022). The current study provides support for families similarly assessing how “sensory-friendly” activities and environments are and gravitating to those that are compatible to their autistic adults’ sensory preferences. It is possible that since family members completed the AASPs, that the corresponding GPS data reflects families planning activities based on their own perceptions of the autistic adult’s sensory preferences. It is unclear, however, if lower rates of participation are indicative of fewer suitable sensory compatible options in the community, or simply the identification of a few preferred activities and locations that are incorporated into one’s routine.

Routines, an integral part of daily life that provide predictability, may be particularly important for autistic adults, due, in part, to their sensory processing patterns. For example, John, whose sensation avoidance was “much more than other people,” structured his day so that he was in the community at less busy times. He also spent much of his time at home, likely because the sensory stimuli were more predictable and under his control than in the community. This finding aligns with Little et al. (2015) who reported that autistic children with more hyperresponsiveness to sensory stimuli tended to participate less in community activities than activities at home. This may be because at home activities were more routinized, and the sensory stimuli are more predictable than activities outside the home environment (Little et al., 2015).

The sense of predictability, however, is not limited to the home environment. For example, Patti, who presented with low registration to sensory stimuli, enjoyed engaging in various community activities as a part of her routine. While people regularly engage in activities at certain places, they may have expectations of the sensory environment, for example, the smells, light, and sound. These expectations become habitual as the person repeatedly engages in the activity and serve as “sensory anchors” that provide a sense of predictability (Bailliard, 2015). Sensory anchors help assure that the activity in which people regularly participate will proceed as anticipated (Bailliard, 2015). For Pete, who visited the same café and ordered the same smoothie for his lunch after work, the smell and the sound of the café in the afternoon and the texture of the smoothie may steer him to maintain these routine behaviors. Overall, autistic adults may choose predictable and controlled environments and participate in activities with which they are familiar to prevent feeling dysregulated or distressed due to unexpected sensory stimuli. In this way, routines may be part of a coping strategy established by the individual or caregiver to manage distress associated with sensory profiles (Crane et al., 2009). Established routines of purposefully chosen, meaningful activities may be an example of a coping strategy developed throughout the lifespan, particularly for those like Steve, John, Pete, and Beth who lived in the same community, and same living situation, for most of their lives.

### Implications for Research or Practice

Environmental factors hold great promise for change and are therefore an attractive target for intervention efforts to increase participation outcomes (Henninger and Taylor, 2012; Tobin et al., 2014). As awareness of the sensory environment’s impact on limiting community activities for autistic individuals has grown, efforts have shifted away from changing the person’s sensory processing to providing more sensory-friendly environments and event days, for example, at museums, movie theaters, and sporting events. These efforts at changing the environment rather than the person have demonstrated some success in improving participation (Fletcher et al., 2018). Results from the current study provide preliminary support that autistic individuals are able to participate in meaningful community activities when individual preferences for both sensory input and desired community activities as well as the sensory demands and opportunities of the environment were considered.

It is important for practitioners to assess sensory processing patterns and educate clients and caregivers about how sensory processing patterns impact participation. Practitioners can assist clients in matching personal sensory processing patterns with activity and environmental demands. Furthermore, practitioners can collaborate with clients to structure routines that facilitate community participation and develop coping strategies for situations when there is a mismatch between one’s sensory profile and environment. It is also critical that efforts aimed at advocating for sensory-friendly environments consider the variety of sensory processing patterns of autistic adults. Ongoing research is needed to further explore the relationship between sensory processing and community participation. This research can contribute to the development of interventions and other initiatives to support the meaningful participation of autistic adults regardless of their sensory processing capacities.

### Limitations

This study included autistic individuals with differing demographic, autistic, and sensory profiles and offers a novel approach to studying sensory processing and community participation. However, the study does have limitations. One limitation is that data were drawn from two studies, neither of which were designed to address sensory processing in detail nor to look specifically at the relationship of sensory processing and community participation. Observation and focused interviewing would add richness to the exploration of sensory processing (Bailliard, 2011). Caregiver and participant interviews designed to address the two constructs together could also yield rich data. Additionally, the AASP was completed by caregivers rather than the autistic adults themselves. Therefore, scores reflect caregivers’ perceptions of their adult child’s sensory processing rather than the adults themselves. Research suggests family members may underestimate sensory impact on daily life (Crane et al., 2009). The data set of six cases examined did not include individuals who scored high in sensation seeking; thus, we did not explore what community participation looks like for autistic adults with this profile. Finally, limited information about the participants’ satisfaction with their community participation was obtained.

## Conclusion

Sensory processing patterns should be considered along with other personal and contextual factors when assessing community participation, both in research and practice. In the current study, those whose sensory processing patterns indicated sensory sensitivity and sensation avoiding described the experience of certain environments and activities as overwhelming and fatiguing and thus either spent less time in the community or visited fewer places compared to those with other sensory processing patterns. Additionally, they utilized adaptive strategies such as structuring daily routines and using sensory anchors. Individuals whose sensory processing patterns were lower in sensory sensitivity and sensation avoiding participated in diverse and preferred activities in their communities. While reviewing the sensory processing and community participation data yielded notable patterns, time and participation in the community, was not the only factor related to sensory processing profiles; other factors such as access to transportation, employment status, finances, and living status appeared to influence time spent in the community and community engagement. Given the importance of community participation for health and wellbeing, further research is needed to understand both the person and contextual factors that support and limit autistic adults’ full participation in community life.

## Data Availability Statement

The raw data supporting the conclusions of this article will be made available by the authors, without undue reservation.

## Ethics Statement

The studies involving human participants were reviewed and approved by the University of North Carolina at Chapel Hill. The participants provided their written informed consent to participate in this study. Written informed consent was obtained from the individual(s) for the publication of any potentially identifiable images or data included in this article.

## Author Contributions

LK, DC, and NB contributed to the study conception and design of the studies. DC, EL, and NB contributed to the acquisition of data. DC, NB, EL, and Y-CS contributed to the analysis and interpretation of data. All authors contributed to the writing of the manuscript. All authors have approved the final version of the manuscript and are accountable for the work described. All authors contributed to the article and approved the submitted version.

## Funding

Study 1 was made possible by grants from Autism Speaks and Foundation of Hope. Study 2 was made possible from funding in part by The Organization for Autism Research, and The University of North Carolina at Chapel Hill Junior Faculty Development Award and the University Research Council. Assistance for this project was provided by the UNC Intellectual and Developmental Disabilities Research Center (NICHD: P50 HD103573; PI: Joseph Piven).

## Conflict of Interest

The authors declare that the research was conducted in the absence of any commercial or financial relationships that could be construed as a potential conflict of interest.

## Publisher’s Note

All claims expressed in this article are solely those of the authors and do not necessarily represent those of their affiliated organizations, or those of the publisher, the editors and the reviewers. Any product that may be evaluated in this article, or claim that may be made by its manufacturer, is not guaranteed or endorsed by the publisher.

## Footnotes

^1^We have chosen to use identity first language (autistic) rather than person-first language (person with autism) given the preferred language of many autistic self-advocates (Bury et al., 2020; Botha et al., 2021).
